# The effect of GLP-1 receptor agonists on autophagy: insights gathered from research evaluating neurodegenerative disorders with these agents

**DOI:** 10.1017/neu.2026.10060

**Published:** 2026-02-13

**Authors:** Maria-Christina Sioufi, Isabela Heroiu, Sabrina Wong, Gia Han Le, Christine E. Dri, Yang Jing Zheng, Kayla M. Teopiz, Taeho Greg Rhee, Heidi Ka Ying Lo, Hernan F. Guillen-Burgos, Roger S. McIntyre

**Affiliations:** 1 Department of Physiology, University of Toronto, Canada; 2 Department of Human Biology, University of Toronto, Canada; 3 Brain and Cognition Discovery Foundation, Canada; 4 Queen’s University, Canada; 5 Department of Pharmacology and Toxicology, University of Toronto, Canada; 6 Institute of Medical Science, University of Toronto, Canada; 7 Poul Hansen Family Centre for Depression, University Health Network, Canada; 8 Department of Psychiatry, Yale School of Medicine, USA; 9 Department of Public Health Sciences, University of Connecticut School of Medicine, USA; 10 Department of Psychiatry, School of Clinical Medicine, LKS Faculty of Medicine, the University of Hong Kong, Hong Kong; 11 Department of Psychiatry and Mental Health, Pontificia Universidad Javeriana, Colombia; 12 Center for Clinical and Translational Research, Colombia; 13 Department of Psychiatry, University of Toronto, Canada

**Keywords:** autophagy, glucagon-Like Peptide-1, Alzheimer’s disease, Parkinson’s disease, GLP-1 Receptor Agonists

## Abstract

**Objective:**

Impaired autophagy has been implicated in the pathophysiology of neurodegenerative disorders, such as Alzheimer’s Disease (AD) and Parkinson’s Disease (PD). Consistent and replicated evidence indicate that Glucagon-like Peptide-1 Receptor Agonists (GLP-1RAs) exert treatment and preventative effects across disparate neurologic and mental disorders, potentially through mechanisms involving autophagy. This systematic review examined the effects of GLP-1RAs on autophagy in cell and animal models of AD and PD, as a proof of concept, to determine if these agents can be repurposed for the prevention and treatment of neurodegenerative and other mental disorders.

**Methods:**

A systematic search on PubMed, Web of Science, and OVID (Medline, Embase, and APA PsycInfo databases) was conducted from inception to June 17, 2025. Screening was performed independently by two reviewers (MCS and IH) using predefined inclusion and exclusion criteria. Subsequently, a quality assessment was conducted.

**Results:**

The search yielded 142 studies, of which 14 were included. Across studies, GLP-1RAs (e.g., liraglutide, semaglutide, and exendin-4) autophagy-specific markers, including beclin-1, LC3-II/LC3-I, ATG7, ATG3, and LAMP1, while normalising p62 levels.

**Discussion:**

In addition to promoting neurogenesis, neuroplasticity, and reducing inflammation, GLP-1RAs appear to modulate molecular and cellular systems contributing to autophagy, potentially mediating their broad therapeutic effects. Collectively, these studies present promising findings of GLP-1RAs for neurodegenerative and mental disorders; however, further studies are required to establish their translatability to human populations.

## Graphical abstract

**Figure:**
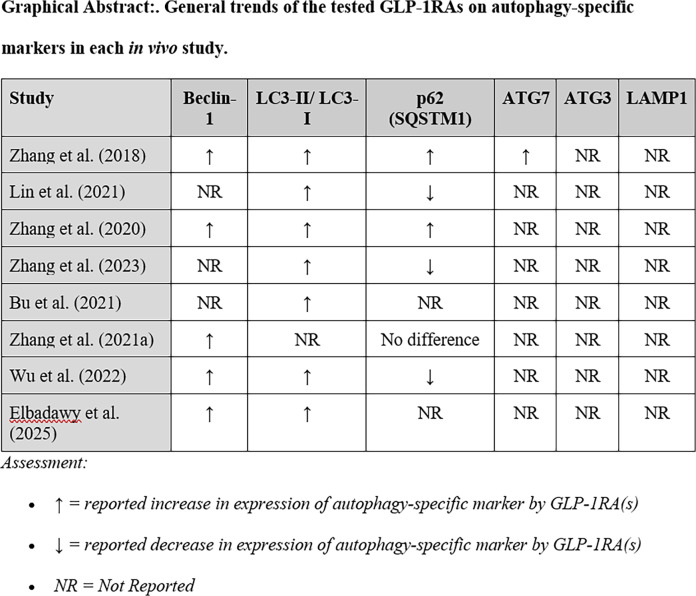

Summations

- Glucagon-like peptide-1 receptor agonists (liraglutide, semaglutide, and exendin-4) were found to restore autophagic flux in both cell and animal models of neurologic disorders.
- Some discrepancies in autophagy-specific marker levels were detected between studies in response to disease induction and intervention administration, potentially due to a lack of assessing dynamic autophagy.

Considerations

- No human studies were included, limiting translatability to human populations.
- The included preclinical studies had varying models, intervention type (liraglutide, semaglutide, or exendin-4), dose, and end points, making direct comparisons challenging.

## Introduction

Alzheimer’s disease (AD) and Parkinson’s disease (PD) are progressive neurodegenerative disorders that negatively impact the lives of those afflicted (Caligiore *et al*., 2022). AD is the most common form of dementia, with an estimated prevalence of 416 million persons worldwide from 2010 to 2020 and growing incidence rates (increasing by 147.95% from 1990 to 2019) (Gustavsson *et al*., 2023; Li *et al*., 2022). AD is primarily characterised by memory impairment and cognitive decline (Alzheimer’s disease facts and figures, 2024). Additionally, hallmark neuropathological features include the presence of extracellular amyloid-beta (a*β*) plaques and intracellular neurofibrillary tangles (NFTs) (DeTure and Dickson, 2019). The accumulation and aggregation of the aforementioned features is associated with impaired synaptic communication, dysfunctional mitochondria, neuroinflammation, and neuronal death, among other complications (Bloom, 2014).

Meanwhile, the global incidence cases of PD in 2021 were approximately 1.34 million, with a prevalence of roughly 11.77 million persons worldwide (a 273.76% increase from 1990) (Luo *et al*., 2024; Li *et al*., 2025). PD is primarily characterised by motor manifestations, including bradykinesia, resting tremor, and muscular rigidity. However, a wide spectrum of non-motor features, such as affective disturbances and cognitive impairments, also constitute core components of the disorder and may exert an equal or even greater impact on patients’ quality of life (Hayes, 2019). Typical pathological characteristics of PD include the loss of dopaminergic neurons in the substantia nigra (SN) and the misfolding and clumping of alpha-synuclein (*α*-syn), which eventually forms Lewy bodies. The aggregation and accumulation of these toxic proteins are thought to contribute to neuroinflammation, disrupted synaptic function, mitochondrial impairment, and cell death (Kim *et al*., 2014; Reich and Savitt, 2019).

In addition to neurodegenerative disorders, chronic mental disorders [e.g., Major Depressive Disorder (MDD) and Bipolar Disorder (BD)] are also associated with substantial morbidity and mortality (Maj *et al*., 2020; McIntyre *et al*., 2020, 2023).

Notably, several studies have demonstrated that the pathophysiology of the aforementioned conditions includes alterations in cellular autophagy (Uddin *et al*., 2018; Nechushtai *et al*., 2023). Autophagy is the regulated process by which intracellular components are delivered to the lysosome to either be degraded or recycled.

There are three types of autophagy, which are primarily characterised by their method of cargo delivery: macro-autophagy, micro-autophagy, and chaperone-mediated autophagy (CMA) (Levine and Kroemer, 2008). Macro-autophagy has received disproportionality greater emphasis in research. Briefly, macro-autophagy (hereafter referred to as autophagy) involves autophagosomes, double-membraned vesicles that are newly created in the cytosol, which ultimately seal cargo inside to be transported and degraded in the lysosome via vesicular fusion. In micro-autophagy, cytoplasmic components are directly internalised via invagination of the lysosomal membrane, forming intraluminal vesicles for breakdown. In contrast, CMA is highly selective: chaperone proteins recognise cytosolic proteins with a targeting motif and translocate them across the lysosomal membrane for degradation (Parzych and Klionsky, 2014; Kaushik and Cuervo, 2018).

Autophagy is triggered by numerous cellular stress stimuli, including but not limited to redox stress, endoplasmic reticulum (ER) stress, mitochondrial damage, nutrient deprivation, hypoxia, and immune signals, resulting in the activation of various pathways coordinated by several autophagy-related gene (ATG) proteins (Kroemer *et al*., 2010; Shibutani *et al*., 2015; Raudenska *et al*., 2021). Any defects in autophagy disrupt cellular homeostasis and can lead to serious consequences potentially linked to neurodegenerative and other mental disorders (Wong and Cuervo, 2010).

Notably, replicated evidence suggests that glucagon-like peptide-1 receptor agonists (GLP-1RAs), originally developed for type 2 diabetes (T2D), may be effective in the treatment and prevention of neurodegenerative and other mental disorders (Au *et al*., 2025b; Lee *et al*., 2025; McIntyre *et al*., 2025; Hölscher, 2024). Their neuroprotective effect may partly be due to the essential role of brain insulin signalling in synaptic plasticity and neuronal survival (Wong *et al*., 2025; Zheng *et al*., 2025). Since brain insulin resistance is commonly found in neurodegenerative disorders, it is plausible that the insulin-sensitising effect of GLP-1RAs could ameliorate this and restore cognitive function (Watson *et al*., 2019; Hong *et al*., 2024; Sun and Mi, 2025). However, beyond this role, GLP-1RAs’ mechanistic basis in the treatment of psychiatric disorders implicates several other systems, with accumulating evidence suggesting direct influence on autophagy (Zhang *et al*., 2021b; Hong *et al*., 2024). The overarching objective herein is to synthesise results of studies that have evaluated the effects of GLP-1RAs on molecular and cellular aspects of autophagy to assess the therapeutic potential of these agents in neurodegenerative disorders. investigate the effect of GLP-1RAs on autophagy and the implications of repurposing these agents as a potential prevention and treatment of neurodegenerative disorders, such as AD and PD.

## Methods

### Search strategy

This study was conducted in accordance with the Preferred Reporting Items for Systematic Reviews and Meta-Analyses (PRISMA) (Page *et al*., 2021). A literature search was performed for studies that investigated the effect of GLP-1RAs on autophagy in models of neurodegenerative disorders, specifically AD and PD.

A systematic search was conducted on PubMed, Web of Science, and OVID (including Medline, Embase, and APA PsycInfo databases) from inception to June 17, 2025 for English-language articles using the following search string: ((‘GLP-1’ OR ‘Glucagon-Like Peptide-1’ OR ‘GLP-1 agonist’ OR ‘GLP-1 receptor agonist’ OR ‘Glucagon-Like Peptide-1 agonist’ OR ‘Glucagon-Like Peptide-1 receptor agonist’ OR ‘Glucagon like peptide-1’ OR ‘Semaglutide’ OR ‘Ozempic’ OR ‘Wegovy’ OR ‘Rybelsus’ OR ‘Dulaglutide’ OR ‘Trulicity’ OR ‘Liraglutide’ OR ‘Saxenda’ OR ‘Victoza’ OR ‘Tirzepatide’ OR ‘Mounjaro’ OR ‘Zepbound’ OR ‘Exenatide’ OR ‘Byetta’ OR ‘Bydureon’ OR ‘Bydureon BCise’) AND (Autophagy) AND (‘Alzheimer’s’ OR ‘Alzheimer’s Disease’ OR ‘Neurodegenerative Disorder’ OR ‘Dementia’ OR’ Parkinson’s’ OR ‘Parkinson’s Disease’ OR ‘Neurological Disorder’ OR ‘Memory Disorder’)).

Subsequently, two reviewers (M.C.S. and I.H.) independently screened the studies on Covidence to prevent any influence on decisions from either of the reviewers and to enable transparency with the screening process. After duplicates were removed, articles were assessed based on their title, abstract, and full text according to the eligibility inclusion/exclusion criteria. Conflicts on screening were resolved via a discussion and a consensus decision.

### Eligibility criteria

Any experimental studies using *in vitro* or *in vivo* models to investigate AD or PD were included. The presence of AD in these models was assessed based on the detection of two core biomarkers: (1) amyloid-beta (a*β*) plaques and (2) NFTs of abnormally phosphorylated tau proteins (Jack *et al*., 2024). Meanwhile, PD models were included based on the presence of key pathological features, including *α*-syn aggregation and the progressive loss of dopaminergic neurons (Ioghen *et al*., 2023; Pandey *et al*., 2024). Furthermore, the presence of key clinical motor symptoms in *in vivo* samples must be observed: bradykinesia in combination with rigidity or resting tremors, or both (Postuma *et al*., 2015).

Studies were included if they investigated the effect of FDA-approved GLP-1RAs in comparison to controls who received either a placebo or no intervention. Additionally, studies that reported any changes in autophagy along with improvements in core symptoms of AD or PD were included.

Studies were excluded if they: (1) did not have participants with AD or PD (i.e., had any other neurodegenerative condition); (2) used non-FDA-approved GLP-1RAs or any form of dipeptidyl peptidase-4 inhibitors due to any potential differences in mechanism from GLP-1RAs; (3) did not assess autophagy as an outcome; (4) had any other study design except an experimental/preclinical or randomised controlled trial (RCT).

### Data extraction and analysis

Data extraction was performed independently by two reviewers (M.C.S. and I.H.) on Google Spreadsheets. The studies were split into two sections: an *in vitro* section and an *in vivo* section. Once in their respective sections, the studies were organised and analysed through several components: (1) author; (2) disease model (AD or PD); (3) study design (experimental vs RCT); (4) cell type used (for *in vitro* studies) or animal model characteristics (for *in vivo* studies); (5) stressor used; (6) sample size; (7) type of GLP-1RA used and its dosage; (8) groups (control(s) and experimental); (9) main findings pertaining to autophagy. To enable some level of comparability between all the studies, a focus on extracting data on autophagy-specific markers such as beclin-1, ATG7, ATG3, microtubule-associated protein light chain 3 (LC3-II/LC3-I), lysosomal-associated membrane protein 1 (LAMP1), and p62 [also commonly known as sequestosome 1 (SQSTM1)] was done. The extracted data were assessed for inclusion by both reviewers.

### Assessment of reporting biases

Risk of bias (RoB) was assessed by two independent reviewers (M.C.S. and I.H.). The SYRCLE’s RoB tool for animal studies was utilised for *in vivo* studies (Hooijmans *et al*., 2014). Meanwhile, the Modified SYRCLE’s RoB tool was employed for *in vitro* studies (De la Rosa González *et al*., 2024). All conflicts were resolved by discussion between the two reviewers.

## Results

### Search results

**Figure 1.**
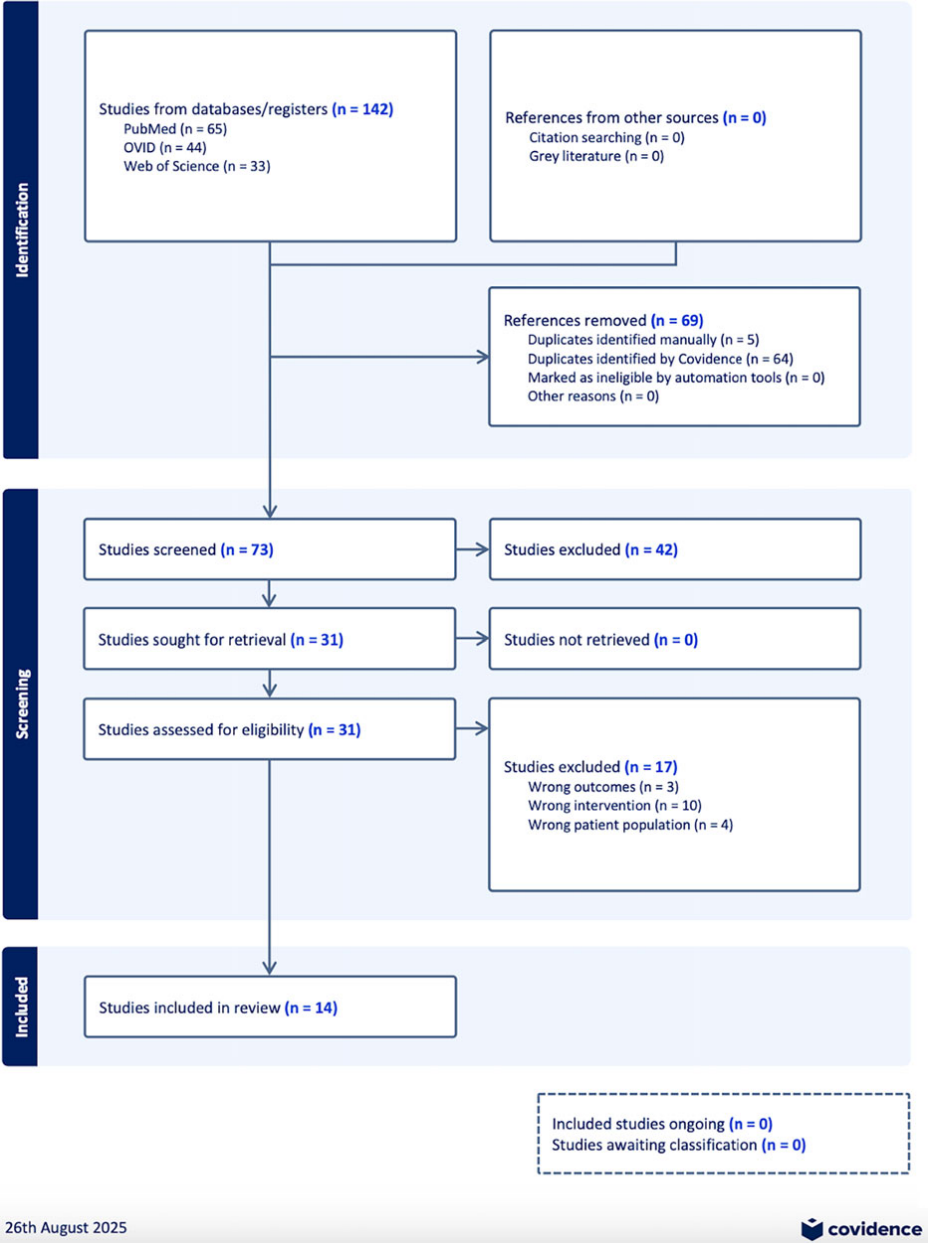
Preferred reporting items for systematic reviews and meta-analyses (PRISMA) flow diagram. The detailed procedure of screening and selecting studies that assess the effect of glucagon-like peptide-1 receptor agonists on autophagy.

The initial literature search yielded 142 studies, of which 64 duplicates were identified on Covidence and 5 were identified manually and removed. Based on title and abstract screening, 42 studies were deemed irrelevant, while the remaining 31 studies progressed onto full-text screening. Seventeen studies were excluded for having either the wrong intervention [e.g., a dual agonist or non-FDA-approved GLP-1RAs (*n* = 10)], incorrect patient population [e.g., samples were not models of AD or PD but of other conditions (*n* = 4)], or wrong outcomes [i.e., did not look at autophagy as an outcome (*n* = 3)]. As a result, 14 articles were included in the review herein and underwent data extraction (Figure 1).

### Study characteristics

Of the total 14 articles included, two studies (Bu *et al*., 2021; Zhang *et al*., 2021a) incorporated both *in vivo* and *in vitro* experiments; therefore, each component was treated as distinct and assessed separately during data extraction. Accordingly, 8 studies were considered *in vivo* RCTs, while the other 8 were *in vitro* experimental studies. Of the *in vitro* cell studies, only 1 used Lund Human Mesencephalic (LUHMES) dopaminergic-like neurons (Panagaki *et al*., 2023) while the rest used the SH-SY5Y human neuroblastoma cell line as their model. For their intervention, a total of 3 studies examined liraglutide (Lira) (Panagaki *et al*., 2017; Kong *et al*., 2020; Panagaki *et al*., 2023), 2 studies investigated exendin-4 (Ex-4) (Bu *et al*., 2021; Zhang *et al*., 2021a), and 1 study explored semaglutide (Sema) (Chang *et al*., 2020); furthermore, 2 studies investigated more than 1 GLP-1RA: 1 study examined Lira and Ex-4 (Jalewa *et al*., 2016), and 1 study looked at Lira and Sema (Liu *et al*., 2022).

From the included *in vivo* studies, only 3 used transgenic models (Bu *et al*., 2021; Zhang *et al*., 2023; Elbadawy *et al*., 2025), while the others primarily utilised environmental stressors such as 1-methyl-4-phenyl-1,2,3,6-tetrahydropyridine (MPTP), 6-hydroxydopamine (6-OHDA), or thapsigargin. For their intervention, a total of 4 studies examined Lira (Zhang *et al*., 2020; Lin *et al*., 2021; Wu *et al*., 2022; Zhang *et al*., 2023), 2 studies investigated Ex-4 (Bu *et al*., 2021; Zhang *et al*., 2021a), 1 study explored Sema (Elbadawy *et al*., 2025), and 1 study looked at both Lira and Sema (Zhang *et al*., 2018).

Overall, 5 articles also included dual agonists as another comparison intervention; however, only results pertaining to FDA-approved single GLP-1RAs were included from these studies (Jalewa *et al*., 2016; Zhang *et al*., 2020; Zhang *et al*., 2021a; Panagaki *et al*., 2023; Zhang *et al*., 2023). A summary of the study characteristics is exhibited in Table 1.

**Table 1.** Study characteristics of in vitro and in vivo studies included

*In vitro* studies					
Study	Disease model	Cell type used	Stressor	GLP-1RA used	Main findings
Jalewa *et al*. (2016)	PD	SH-SY5Y	Rotenone (1 µM)	1. Lira (100 nM) 2. Ex-4 (100 nM)	– Lira increased expression of ATG7 (*p* < 0.01), ATG3, and LC3A/B by 28.3%, 39.5%, and 34.5%, respectively, compared to control unstressed cells – Ex-4 increased ATG7 expression by 19.2% compared to control cells
			LY294002 (50 µM)		– Lira increased ATG3 expression by 1.8-fold (*p* < 0.0001) compared to stressed control cells – Ex-4 did not ameliorate reduced ATG3 expression in the stressed group compared to control cells
Kong *et al*. (2020)	AD	SH-SY5Y	Overexpressed APP695swe plasmid	Lira (10 nM)	Lira significantly increased LC3II/LC3I ratio (1.259-fold) and beclin-1 levels (1.238-fold), and significantly reduced p62 levels (0.852-fold) compared to APP control (*p* < 0.05)
Bu *et al*. (2021)	PD	SH-SY5Y	Overexpressed hA53T-*α*-syn	Ex-4 (100 nM)	Ex-4 significantly reduced p62 levels (*p* < 0.05); furthermore, it increased LAMP1 (*p* < 0.0001), beclin-1 (*p* < 0.05), and LC3B-II/LC3B-I) (*p* < 0.0001) compared to control unstressed cells.
Chang *et al*. (2020)	AD	SH-SY5Y	A*β*25–35 (20 µM)	Sema (10 nM)	Sema significantly increased expression of p62 (24.08 ± 1.56%), ATG7 (24.6 ± 2.43%), LC3-II (15.6 ± 2.46), and beclin-1 (23.84 ± 1.997) in A*β*25–35 cells compared to A*β*25–35 cells that did not receive treatment
Liu *et al*. (2022)	PD	SH-SY5Y	6-OHDA (75 µM)	1. Lira (10 nM) 2. Sema (10 nM)	– In the 6-OHDA + Lira group, p62 levels were significantly reduced (*p* < 0.001) by 153.1%±8.8%, while levels of LC3-II/LC3-I (*p* < 0.01), beclin-1 (*p* < 0.001), and ATG7 (*p* < 0.001) significantly increased by 72.8% ± 3.9%, 65.97% ± 6.7%, and 60.2% ± 5.7%, respectively, compared to 6-OHDA cells that did not receive treatment. – In the 6-OHDA + Sema group, LC3-II/LC3-I (84.5% ± 3.6%), beclin-1 (80.6% ± 5.5%), and ATG7 (74.3% ± 5.5%) levels were significantly elevated (*p* < 0.001), while p62 levels (136.6% ± 12.5%) were significantly reduced (*p* < 0.001) compared to untreated 6-OHDA cells – The 6-OHDA + Sema group was more effective in restoring autophagy compared to the 6-OHDA + Lira group. Sema significantly increased LC3-II/LC3-I(*p* < 0.01), beclin-1 (*p* < 0.01), and ATG7 (*p* < 0.05) levels compared to Lira in 6-OHDA treated cells
Panagaki *et al*. (2017)	AD and PD	SH-SY5Y	Thapsigargin (100 nM)	Lira (100 nM)	Lira significantly increased beclin-1 (*p* ≤ 0.001), ATG3 (*p* ≤ 0.01), and LC3 (*p* ≤ 0.05) levels compared to the thapsigargin-only group. ATG7 levels were also increased (*p* ≤ 0.05), but not to a significant extent when examined in a post-hoc analysis.
Panagaki *et al*. (2023)	PD	LUHMES dopaminergic-like neurons	Thapsigargin (100 nM)	Lira (100 nM)	Lira treatment significantly restored the suppressed expression of beclin-1 (*p* ≤ 0.001), ATG3 (*p* = 0.0001), ATG7 (*p* ≤ 0.001), and LC3B (*p* = 0.004) in thapsigargin-treated cells compared to thapsigarginstressed neurons that did not receive treatment.
Zhang *et al*. (2021a)	PD	SH-SY5Y	6-OHDA (350 µM)	Ex-4 (100 nM)	Ex-4 upregulated p62 (*p* < 0.001) and beclin-1 (*p* < 0.01) expression in 6-OHDA-treated cells compared to control cells

*In vitro* studies					
Study	Disease model	Animal model characteristics	Stressor	GLP-1RA used	Main findings
Zhang *et al*. (2018)	PD	Male C57BL/6 mice (10 weeks old; 20-25g; *n* = 12 per group)	MPTP (20 mg/kg)	1. Sema (25 nmol/kg) 2. Lira (25 nmol/kg)	– MPTP + Lira treatment alleviated the impairments of beclin-1 (*p* < 0.01), ATG7 (*p* < 0.05), LC3 (*p* < 0.001), and p62 (*p* < 0.001) expression in the SN compared to the MPTP-only group – MPTP + Sema treatment also restored the reductions of beclin-1, ATG7, LC3, and p62 expression compared to the MPTP-only group (*p* < 0.001) – Sema was more effective at normalising expression of beclin-1 (*p* < 0.05), ATG7 (*p* < 0.001), LC3 (*p* < 0.01), and p62 (*p* < 0.001) compared to Lira in MPTP treated mice
Bu *et al*. (2021)	PD	Female Sprague-Dawley rats (3 months old; 250-280g; *n* = 4)	AAV-9-A53T-*α*-syn injected into the SNpc	Ex-4 (5 μg/kg)	AAV-9-A53T-*α*-syn + Ex-4 significantly increased LC3-II/LC3-I levels (*p* < 0.01) compared to controls that received a placebo (AAV-vehicle with normal saline)
Elbadawy *et al*. (2025)	AD	Male and female transgenic mice (18 weeks old; 22-28g; *n* = 10 per group)	P301S	Sema (25 nmol/kg)	Semaglutide treatment significantly reversed the effects of P301S on autophagy by upregulating the hippocampal content of both beclin-1 and LC3-II (*p* < 0.001)
Lin *et al*. (2021)	PD	Male C57BL/6 mice (8 weeks old; 22-25g; *n* = 3 per group)	MPTP (20 mg/kg)	Lira (Victoza; 0.15 mg/kg)	– MPTP mice treated with Lira were observed to have a non-significant increase in LC3-II/LC3-I (0.76 ± 0.10) in their SN compared to untreated MPTP mice. Furthermore, the addition of Lira to MPTP mice significantly decreased p62 levels (0.69 ± 0.14-fold) compared to the MPTP-only mice (*p* < 0.01) and control mice (*p* < 0.05)
Wu *et al*. (2022)	PD	Male C57BL/6 mice (20 ± 2g; *n* = 3 per group)	MPTP	Lira [either treated with low (25nmol/kg), medium (50nmol/kg), or high (100 mol/kg) dose]	– After treatment with a low, medium, or high dose of Lira, there was a significant increase in LC3II/LC3I (*p* = 0.004, 0.002, and 0.001, respectively) and beclin-1 levels (*p* = 0.014, 0.004, and 0.001, respectively) compared to the MPTP-only group; meanwhile, the p62 levels significantly decreased (*p* = 0.155, 0.009, and 0.001 respectively) compared to the MPTP-only group – The reduction of p62 expression by the high dose of Lira was also statistically significant when compared to the control group (*p* < 0.05) and the MPTP + low dose of Lira group (*p* < 0.05)
Zhang *et al*. (2021a)	PD	Male Sprague-Dawley rats (8 weeks old; 220-250g; *n* = 10 per group)	6-OHDA (5 µL, 2 mg/mL injections)	Ex-4 (10 nmol/kg)	– Ex-4 treatment enhanced beclin-1 expression in dopaminergic neurons within the SN compared to the control group (*p* < 0.05) and the 6-OHDA only group (*p* < 0.05) – There was no significant difference in p62 expression in 6-OHDA mice treated with Ex-4 compared to the control and 6-OHDA-only groups
Zhang *et al*. (2020)	PD	Male C57BL6/J mice (8 weeks old; 20-22g; *n* = 4 per group)	MPTP (25 mg/kg)	Lira (25 mg/kg)	– Lira treatment of MPTP-stressed mice restored beclin-1 (*p* < 0.01) and LC3 (*p* < 0.001) expression to 90.6% and 91.6%, respectively, compared to untreated MPTP-stressed mice. Meanwhile, p62 expression in the dopaminergic neurons of MPTP-stressed mice treated with Lira was downregulated to 85.3% (*p* < 0.05) compared to the control group, but normalisedcompared to untreated MPTP-stressed mice (*p* < 0.001)
Zhang *et al*. (2023)	PD	Male and female A53T transgenic mice (13-14 months old; *n* = 5-6 per group)	A53Thomozygous (+/+) mice	Lira (25 or 50 nmol/kg)	– A53T mice treated with Lira had normalised LC3-II/LC3-1 levels (though not significantly) within their SN compared to the A53T group that received a placebo (saline). Lira treatment also normalised p62 levels by reducing them (*p* < 0.001) compared to the A53T mice that received a placebo.

### Risk of bias assessment

Details of the RoB assessment are presented in Tables 2 and 3 for *in vivo* and *in vitro* studies, respectively. In the majority of the *in vivo* studies, a high risk of selection and performance bias was due to a lack of randomisation and blinding of investigators to intervention groups. Additionally, an unclear RoB was mainly given in circumstances where the criteria of the domain were partially met but not enough to ensure a low RoB. It must be noted that, although all the studies except one (Elbadawy *et al*., 2025) did not have blinded outcome assessors (D7), this was not deemed a serious concern in terms of bias, as the animals were euthanised to measure the outcome related to autophagy. Furthermore, regarding whether the assessment of incomplete data was adequately addressed (D8), only sections related to autophagy were examined for this domain, since only data related to autophagy were extracted.

**Table 2.** *In vivo* risk of bias assessment

Source	D1	D2	D3	D4	D5	D6	D7	D8	D9	D10
Zhang *et al*. (2018)	U	Y	U	Y	N	U	Y	Y	Y	Y
Bu *et al*. (2021)	Y	Y	Y	U	N	U	Y	Y	Y	Y
Elbadawy *et al*. (2025)	N	Y	U	Y	Y	U	Y	Y	Y	Y
Lin *et al*. (2021)	U	Y	U	Y	N	Y	Y	Y	Y	Y
Wu *et al*. (2022)	U	Y	U	Y	N	U	Y	Y	Y	Y
Zhang *et al*. (2021a)	U	Y	U	Y	N	U	Y	Y	Y	Y
Zhang *et al*. (2020)	U	Y	U	Y	U	U	Y	Y	Y	Y
Zhang *et al*. (2023)	N	Y	U	Y	N	U	Y	Y	Y	Y

Risk of bias domains: D1: Selection bias - Sequence generation; D2: Selection bias - Baseline characteristics; D3: Selection bias - Allocation concealment; D4: Performance bias - Random housing; D5: Performance bias - Blinding; D6: Detection bias - Random outcome assessment; D7: Detection bias - Blinding; D8: Attrition bias - Incomplete outcome data; D9: Reporting bias - Selective outcome reporting; D10: Other - Other sources of bias.

Judgment: Yes (Y) - low risk of bias; Unclear (U) - unclear risk of bias; No (N) - high risk of bias.

**Table 3.** *In vivo* risk of bias assessment

Source	D1	D2	D3	D4	D5	D6	D7	D8	D9	D10
Jalewa, *et al*. (2016)	Y	Y	Y	Y	Y	Y	N	Y	U	U
Kong, *et al*. (2020)	N	Y	Y	Y	Y	Y	N	Y	Y	Y
Bu, *et al*. (2021)	Y	Y	N	U	Y	Y	N	Y	U	U
Chang, *et al*. (2020)	N	Y	Y	Y	Y	Y	N	Y	Y	Y
Liu, *et al*. (2022)	N	Y	Y	Y	Y	Y	N	Y	Y	Y
Panagaki, *et al*. (2017)	Y	Y	Y	Y	Y	Y	Y	Y	Y	U
Panagaki, *et al*. (2023)	Y	Y	Y	Y	Y	Y	Y	Y	Y	U
Zhang, *et al*. (2021a)	U	Y	Y	Y	U	Y	N	Y	Y	Y

Risk of bias domains: D1: Selection bias - Sample size calculation; D2: Selection bias - Baseline characteristics; D3: Performance bias - Detailed explanation of intervention; D4: Performance bias - Detailed explanation of culture conditions; D5: Detection bias - Details of comparison group; D6: Detection bias - Method of measurement outcome; D7: Detection bias - Blinding; D8: Attrition bias - Incomplete outcome data; D9: Reporting bias - Selective outcome reporting; D10: Other - Other sources of bias.

*Judgment:* Yes (Y) - low risk of bias; Unclear (U) - unclear risk of bias; No (N) - high risk of bias.

As for the *in vitro* studies, a high risk of detection bias (D7) was due to a lack of blinding of the outcome assessor; moreover, an unclear RoB for other sources of bias (D10) was given to studies that had potentially concerning conflicts of interest, among other reasons.

### Results of individual studies

#### In vivo autophagy-specific biomarkers

Investigations conducted by Zhang *et al*. (2018); Lin *et al*. (2021); Wu *et al*. (2022); and Zhang *et al*. (2020) administered MPTP to male C57BL/6 mice to induce PD-like stress.

Zhang *et al*. (2018) observed a significant suppression of autophagy markers, beclin-1, ATG7, LC3, and p62, in the SN of MPTP-treated mice compared to the control group treated with normal saline (*p* < 0.001) (*n = 12* per group). Administration of Lira to MPTP-treated mice was reported to restore levels of beclin-1 (*p* < 0.01), ATG7 (*p* < 0.05), LC3 (*p* < 0.001), and p62 (*p* < 0.001), compared to the MPTP-only group. Likewise, Sema treatment also alleviated the levels of these autophagy-specific markers in MPTP-treated mice (*p* < 0.001). Overall, Sema was more effective at normalising the expression of beclin-1 (*p* < 0.05), ATG7 (*p* < 0.001), LC3 (*p* < 0.01), and p62 (*p* < 0.001) compared to MPTP mice treated with Lira.

Lin *et al*. (2021) reported a significant reduction of LC3-II/LC3-I (*p* < 0.001) and increased p62 accumulation (*p* < 0.05) in the SN of MPTP-treated mice compared to control mice that received saline (*n = 3* per group). Although mice that only received Lira treatment had significantly increased LC3-II/LC3-I (*p* < 0.05) compared to control mice, MPTP mice treated with Lira were observed to have a non-significant increase in LC3-II/LC3-I compared to MPTP-only treated mice. Mice that only received Lira treatment also had reduced p62 levels compared to control mice (*p* < 0.01). Furthermore, MPTP-mice treated with Lira had significantly decreased p62 levels compared to control (*p* < 0.05) and MPTP-only treated mice (*p* < 0.01).

Wu *et al*. (2022) found a significant decrease in beclin-1 (*p* = 0.006) and LC3-II/LC3-I (*p* = 0.016) in MPTP-treated mice, as well as a significant increase in p62 (*p* = 0.051), compared to control mice that only received a saline injection (*n =* 3 per group). Upon treatment with either a low (25 nmol/kg), medium (50 nmol/kg), or high (100 nmol/kg) dose of Lira, beclin-1 (*p* = 0.014, 0.004, and 0.001, respectively) and LC3-II/LC3-I (*p* = 0.004, 0.002, and 0.001, respectively) were significantly increased, while p62 levels were significantly reduced (*p* = 0.155, 0.009, and 0.001, respectively) compared to MPTP-only treated mice. Additionally, the high dose of Lira was found to be more effective at reducing p62 levels compared to the control mice (*p* < 0.05) and MPTP-treated mice that received the low dose of Lira (*p* < 0.05).

Zhang *et al*. (2020) reported reduced beclin-1 (*p* < 0.01), LC3 (*p* < 0.01), and p62 (*p* < 0.001) levels in the SN of MPTP-treated mice compared to control mice that received saline (*n = 4* per group). MPTP mice treated with Lira were observed to have restored expression of beclin-1 (*p* < 0.01), LC3 (*p* < 0.001), and p62 (*p* < 0.001) compared to untreated MPTP mice.

Studies Bu *et al*. (2021) and Zhang *et al*. (2023) utilised transgenic mice to model PD, while Elbadawy *et al*. (2025) used transgenic mice to induce AD-like stress.

Zhang *et al*. (2023) investigated the effects of Lira in male and female A53T homozygous mice (*n = 5–6* per group). A53T (+/+) mice overexpressed the human version of *α*-syn and were compared to control A53T (–/–) mice that received normal saline. A53T (+/+) mice that received normal saline were observed to have significantly lower levels of LC3-II/LC3-I (*p* < 0.001) and higher levels of p62 (*p* < 0.05) in the SN compared to control mice. A53T (+/+) mice that received Lira treatment were reported to have restored LC3-II/LC3-I levels, though not significantly, compared to saline-treated A53T (+/+) mice. Lira treatment also normalised p62 protein levels by reducing them compared to A53T (+/+) mice that received normal saline (*p* < 0.001).

Bu *et al*. (2021) injected AAV-9-A53T-*α*-syn into the right substantia nigra pars compacta (SNpc) of female Sprague-Dawley rats (*n = 4*). Treatment of these rats with Ex-4 significantly increased the LC3-II/LC3-I ratio (*p* < 0.01) compared to rats that received a placebo (AAV-vehicle and normal saline).

Elbadawy *et al*. (2025) utilised male and female P301S mice (*n = 20*) as a model of taupathy. P301S mice treated with saline (*n = 10*) were found to have significantly reduced hippocampal levels of beclin-1 and LC3-II compared to control C57BL/6 wild-type mice that received saline (*n = 10*) (*p* < 0.001). However, P301S mice treated with Sema (*n = 10*) had upregulated hippocampal content of both beclin-1 and LC3-II compared to P301S mice that received saline (*p* < 0.001).

Zhang *et al*. (2021a) induced PD-like stress in male Sprague-Dawley rats (*n = 10* per group) via 6-OHDA injections. Treatment of 6-OHDA mice with Ex-4 enhanced beclin-1 expression in dopaminergic neurons within the SN compared to a sham group of mice that received saline injections (*p* < 0.05) and untreated 6-OHDA mice (*p* < 0.05). However, there was no significant effect of Ex-4 on p62 expression in 6-OHDA-treated mice compared to the sham and untreated 6-OHDA mice.

The general influence of the tested GLP-1RAs on autophagy-specific markers, reported in each *in vivo* study, is summarised in Table 4.

**Table 4.** General trends of the tested GLP-1RAs on autophagy-specific markers in each *in vivo* study

Study	Beclin-1	LC3-II/LC3-I	p62 (SQSTM1)	ATG7	ATG3	LAMP1
Zhang *et al*. (2018)	↑	↑	↑	↑	NR	NR
Lin *et al*. (2021)	NR	↑	↓	NR	NR	NR
Zhang *et al*. (2020)	↑	↑	↑	NR	NR	NR
Zhang *et al*. (2023)	NR	↑	↓	NR	NR	NR
Bu *et al*. (2021)	NR	↑	NR	NR	NR	NR
Zhang *et al*. (2021a)	↑	NR	No difference	NR	NR	NR
Wu *et al*. (2022)	↑	↑	↓	NR	NR	NR
Elbadawy *et al*. (2025)	↑	↑	NR	NR	NR	NR

Assessment: ↑, reported increase in expression of autophagy-specific marker by GLP-1RA(s); ↓, reported decrease in expression of autophagy-specific marker by GLP-1RA(s); NR, not reported.

#### In vitro autophagy-specific biomarkers

Studies Zhang *et al*. (2021a) and Liu *et al*. (2022) treated SH-SY5Y cells with 6-OHDA as a model of PD.

Liu *et al*. (2022) reported a significant reduction in beclin-1, ATG7, and LC3-II/LC3-I, as well as increased p62 levels, in 6-OHDA-treated cells compared to the control group (*p* < 0.001). Treatment of 6-OHDA cells with Lira was observed to significantly enhance levels of beclin-1 (*p* < 0.001), ATG7 (*p* < 0.001), and LC3-II/LC3-I (*p* < 0.01), while significantly reducing levels of p62 (*p* < 0.001) compared to cells that only received 6-OHDA. Meanwhile, 6-OHDA cells treated with Sema were found to have elevated levels of beclin-1, ATG7, and LC3-II/LC3-I compared to untreated 6-OHDA cells (*p* < 0.001); furthermore, Sema treatment decreased levels of p62 compared to the untreated 6-OHDA cells (*p* < 0.001).

Zhang *et al*. (2021a) reported that 6-OHDA cells treated with Ex-4 had upregulated p62 expression compared to the control group (*p* < 0.001) and untreated 6-OHDA cells (*p* < 0.01). Furthermore, Ex-4 treatment was observed to also enhance beclin-1 expression levels compared to the control group (*p* < 0.01) and untreated 6-OHDA cells (*p* < 0.05).

Studies Panagaki *et al*. (2017) and Panagaki *et al*. (2023) utilised a thapsigargin, a sarcoplasmic reticulum ATPase inhibitor to induce stress.

Panagaki *et al*. (2023) used LUMES dopaminergic-like neurons as a model of PD. Neurons treated with thapsigargin had reduced neuronal expression of autophagy-specific markers, beclin-1, ATG3, ATG7, and LC3B, compared to the control (*p* ≤ 0.001). However, Lira treatment restored the suppressed expression of these markers compared to thapsigargin-only stressed neurons (*p* ≤ 0.001).

Panagaki *et al*. (2017) utilised SH-SY5Y cells as a general model of PD and AD. When treated with thapsigargin, the cells had significantly suppressed expression of beclin-1 (*p* ≤ 0.001), ATG3 (*p* ≤ 0.01), ATG7 (*p* ≤ 0.001), and LC3 (*p* ≤ 0.001) compared to the control group. However, Lira treatment of these cells elevated levels of beclin-1 (*p* ≤ 0.001), ATG3 (*p* ≤ 0.01), ATG7, and LC3 (*p* ≤ 0.05) compared to thapsigargin-only treated cells.

Jalewa *et al*. (2016) administered Lira or Ex-4 to SH-SY5Y cells with rotenone-induced autophagy impairment. Upon administration of Lira, a significant increase in ATG7 levels compared to non-stressed SH-SY5Y controls was observed (*p* < 0.01). Treatment with Ex-4 also increased ATG7 expression, although it was not statistically significant. Moreover, Lira increased the expression of ATG3 and LC3A/B compared to non-stressed SH-SY5Y controls. In another experiment, Jalewa *et al*. (2016) exposed SH-SY5Y cells to LY294002, a phosphoinositide 3-kinase (PI3K) inhibitor, followed by either Lira or Ex-4 treatment. Lira treatment enhanced ATG3 expression levels (*p* < 0.0001), whereas Ex-4 did not ameliorate ATG3 expression compared to the stressed SH-SY5Y controls.

Bu *et al*. (2021) overexpressed hA53T-*α*-syn, a mutated form of *α*-syn, in SH-SY5Y cells as a model of PD. Treatment with Ex-4 was reported to significantly enhance autophagic flux by increasing expression of LAMP1 (*p* < 0.0001), beclin-1 (*p* < 0.05), and LC3B-II/LC3B-I ratio (*p* < 0.0001), as well as by decreasing p62 levels compared to the control group that received a phosphate-buffered saline pre-treatment.

Chang *et al*. (2020) treated SH-SY5Y cells with a*β*_25–35_ as a model of AD, which was reported to significantly reduce beclin-1, LC3-II, ATG7, and p62 levels compared to the control (*p* < 0.001). Sema treatment appeared to reverse the damaging effects of a*β*_25–35_ on autophagy by normalising the expression of beclin-1, LC3-II, ATG7, and p62 compared to untreated a*β*_25–35_ cells (*p* < 0.001).

Kong *et al*. (2020) transfected and overexpressed APP695swe plasmid, a mutated human amyloid precursor protein (APP) known to overproduce a*β* proteins, in SH-SY5Y cells as a model of AD. Treatment with Lira significantly increased beclin-1 and LC3-II/LC3-I compared to APP695swe/SH-SY5Y controls (*p* < 0.05). Lira treatment also significantly reduced expression of p62 compared to APP695swe/SH-SY5Y controls (*p* < 0.05).

The general influence of the tested GLP-1RAs on autophagy-specific markers, reported in each *in vitro* study, is summarised in Table 5.

**Table 5.** General trends of the tested GLP-1RAs on autophagy-specific markers in each *in vitro* study

Study	Beclin-1	LC3-II/ LC3-I	p62 (SQSTM1)	ATG7	ATG3	LAMP1
Jalewa *et al*., (2016)	NR	↑	NR	↑	↑	NR
Bu *et al*. (2021)	↑	↑	↓	NR	NR	↑
Liu *et al*. (2022)	↑	↑	↓	↑	NR	NR
Zhang *et al*. (2021a)	↑	NR	↑	NR	NR	NR
Panagaki *et al*. (2023)	↑	↑	NR	↑	↑	NR
Panagaki *et al*. (2017)	↑	↑	NR	↑	↑	NR
Chang *et al*. (2020)	↑	↑	↑	↑	NR	NR
Kong *et al*. (2020)	↑	↑	↓	NR	NR	NR

Assessment: ↑, reported increase in expression of autophagy-specific marker by GLP-1RA(s); ↓, reported decrease in expression of autophagy-specific marker by GLP-1RA(s); NR, not reported.

## Discussion

This systematic review investigating the effect of GLP-1RAs on molecular and cellular systems salient to autophagy found a consistent and highly replicated restoration and enhancement of autophagy in response to GLP-1RA treatment in cell and animal models of neurological disorders (e.g., AD and PD). Collectively, the *in vitro* and *in vivo* studies reported elevated levels of key autophagy-specific markers, such as beclin-1, LC3-II/LC3-I, ATG7, ATG3, and LAMP1, as well as reduced p62 levels, indicating augmented autophagic flux.

### Autophagy-specific markers

Among some of its other functions, beclin-1 is a central regulator of autophagy. Beclin-1 serves as a scaffold protein that forms an autophagy-specific complex with multiple other proteins at the ER to create a phagophore, the precursor of an autophagosome, ultimately initiating autophagy (Tran *et al*., 2021). Deficiency of beclin-1 in response to neurodegenerative-induced stress was consistently observed among the included studies and is supported by other literature (Pickford *et al*., 2008). Moreover, an elevation of beclin-1 after GLP-1RA administration was a common finding, indicating restored or elevated autophagy.

LC3-I is a cytosolic protein that is conjugated to phosphatidyl-ethanolamine (PE) to form LC3-II. The lipidation of LC3-I to LC3-II enables its insertion into the autophagosome inner and outer membranes, serving as a crucial marker of autophagosomes (Ye *et al*., 2023; Runwal *et al*., 2019). The included studies reported a notable reduction in the ratio of LC3-II/LC3-I after AD and PD-like stress was induced, indicating impaired autophagy. Following GLP-1RA treatment, LC3-II levels were rescued, implying restored autophagic flux.

ATG7 and ATG3 act as E1-like and E2-like enzymes, respectively. The aforementioned proteins are integral to the process of catalysing the conjugation of LC3-I to PE to form LC3-II after autophagy has been induced (Frudd *et al*., 2018). To our knowledge, Zhang *et al*. (2018) was the only *in vivo* study to explore the impact of neurodegenerative stress and GLP-1RA on ATG7, while several other *in vitro* studies investigated both ATG7 and ATG3 levels. The collective finding was a suppression of both ATG7 and ATG3 levels after disease induction, with an increase of these markers after GLP-1RA treatment.

In addition to its other roles, p62 functions as a receptor that binds specific ubiquitin-tagged proteins and damaged organelles, delivering them to the autophagosome for degradation. Since p62 is degraded along with the cargo, accumulated levels of p62 are thought to be indicative of impaired autophagy (Liu *et al*., 2016; Kumar *et al*., 2022). There were conflicting findings within *in vitro* and *in vivo* studies regarding the influence of neurodegenerative-induced stress and GLP-1RAs on p62 levels. Some studies reported elevated p62 in response to disease induction, with GLP-1RAs reducing it. Other articles reported the opposite, where AD and PD lowered p62 levels, and GLP-1RA treatment restored them. Despite this discrepancy, the majority of the included studies, as well as other literature, support the former, where neurodegenerative stress raised p62 levels, with subsequent normalisation of p62 levels following GLP-1RA administration (Runwal *et al*., 2019; Bjørkøy *et al*., 2009).

LAMP1 is a glycosylated transmembrane protein found in lysosomes (Cheng *et al*., 2018). The literature delineates that disruption of LAMP1 is associated with an accumulation of autophagy vesicles as a result of ineffective clearance inside lysosomes (Eskelinen, 2006). Bu *et al*. (2021) was the only study to investigate this lysosomal marker and found a notable increase in LAMP1 levels following Ex-4 treatment.

### Limitations

While these findings yield valuable insights into GLP-1RAs’ modulation of autophagic processes, there are some limitations to consider, such as the heterogeneity of the included studies. The data reported in this review was collected from preclinical studies that utilised a variety of different cell and animal models, GLP-1RAs (types and dosages), and end points, which made direct comparisons difficult. Moreover, a non-standardised tool was employed to assess the risk of bias for the *in vitro* studies. None of the studies that we were reviewing were clinical studies, which represents a very significant limitation, and the extent to which the preclinical findings extend to human results is uncertain.

Furthermore, relatively few studies assessed dynamic autophagic flux. As such, deciphering marker levels in association with improved or impaired autophagy can be misconstrued when the timing and context aren’t considered. This limitation could explain the incongruence in p62 levels after disease induction and GLP-1RA administration, as other articles have reported that p62 levels are not always inversely associated with autophagic flux during cellular stress (Liu *et al*., 2016). Another instance is the interpretation of LC3-II/LC3-I levels, which can also be somewhat problematic, as LC3-II levels measured at a single time point do not necessarily reflect overall autophagic flux (Mizushima and Yoshimori, 2007). For instance, elevations in LC3-II could indicate enhanced autophagosome formation through increased conjugation of LC3-I with PE; however, it could also imply ineffective and substandard lysosomal degradation (leading to an accumulation of autophagosomes with LC3-II) (Sarkar *et al*., 2014; Sharifi *et al*., 2015). It remains a possibility that the discrepancies and/or inconsistencies that we have identified in the markers of autophagy that we have evaluated may be related to the inadequate assessment of dynamic autophagy processes as much of the datapoints could be considered static, it would be the case that a more comprehensive and dynamic autophagy approach may result in findings that are discrepant from what we have observed.

An overarching limitation which emanates and surrounds the entire literature review that we have conducted relates to the heterogenous findings. It would be accurate to state that our findings represent a disparate assortment of molecular readouts, most of which are in need of replication especially in human subjects. The degree to which the effects we are observing are specific or pseudospecific cannot be ascertained from the methodologic approach we have taken. Notwithstanding this overarching limitation, a theme throughout the results that is relatively consistent is the observation that molecular changes that are observed relate to processes integral to autophagy.

### Future directions

Future research could assess the dynamic process of autophagy, as it may provide a clearer and more integrative understanding of what is occurring, as opposed to static measurements. Additionally, future studies should broaden their investigation to examine the influence of GLP-1RAs on other neurodegenerative conditions and mental disorders. Performing clinical trials would also prove valuable, as they could evaluate whether these findings translate to human populations.

Recent research has investigated microbiota-based therapeutics in ameliorating autophagy through modulating endogenous GLP-1 as a potential method to mediate AD and PD symptomology (Bonfili *et al*., 2017; Qi *et al*., 2025). Further investigation of this approach could reinforce understanding of these relationships. Another avenue of exploration is the influence of dual agonists on autophagy as opposed to single GLP-1RAs, as the dual agonists have also been reported to have a potentially more robust effect on markers of autophagy (Jalewa *et al*., 2017; Panagaki *et al*., 2018; Cai *et al*., 2021).

## Conclusion

Collectively, this systematic review found that GLP-1RAs enhance autophagy through augmenting and normalising key autophagy-specific markers, accompanied by improvements in AD and PD symptoms. These findings suggest that GLP-1RAs may exert therapeutic effects in neurologic disorders through autophagy along with other highly replicated impacts on plasticity, trophism, and neuroprotection. Future research should expand these findings to further evaluate the potential of repurposing GLP-1RAs for the prevention and treatment of other neurologic and mental disorders.
